# Faculty of Comparative Medicine—McGill University—Commencement

**Published:** 1892-04

**Authors:** 


### FACULTY OF COMPARATIVE MEDICINE—McGILL UNIVERSITY—COMMENCEMENT.

The Wm. Molson Hall, McGill University, was the scene on Thursday, March 31st, of a most interesting and brilliant gathering, the annual convocation of the Faculty of Comparative Medicine. A large number of friends and relatives of the graduate sophomores and freshmen were in attendance, together with a large number of "under-goods" to do honor to the graduating class of 1892.

Mr. J. H. R. Molson took the chair, and was supported by Vice-Chancellor Sir Wm. Dawson and Mr. W. C. McDonald. Among others on the platform were: the Dean, Dr. Duncan, Mr. Eachran, Dr. C. McEachran, Dr. Bryden, Boston; Dr. Gadsden, Philadelphia; Dr. Craik, Professor Mills, J. Redpath Dougall, Rev. Geo. Cornish, L. L. D.; S. Finlay, Prof. McLeod, H. Watt and J. W. Brakenridge, B. C. L.

The dean of the faculty, Dr. D. McEachran, read the twenty-sixth annual report of the faculty and prize lists. He said :

"In presenting this, the twenty-sixth annual report at this third convocation as a faculty of the University, I have much pleasure in stating that the efforts of the faculty in endeavoring to keep up the standard of education in this particular branch of professional training, continue to merit and receive the public confidence, as is evidenced by students being attracted from no less than nine States of the Union and every province in Canada.

He noted: "The success of their graduates who are practicing and teaching, many of them filling with much credit, positions of responsibility as lecturers and quarantine officers, not a few doing excellent work in original research of value, not only to their own profession but to medical science at large. The importance of the study of comparative medicine is daily becoming more understood and appreciated by thinking people in and out of the professions, and no medical college can be considered complete unless its curriculum embraces a thorough course of comparative pathology.

"So closely related to one another are human and animal diseases, so frequently does the one originate from the other, that a graduate in medicine or a graduate in comparative cannot be considered to have completed his education till he has acquired an intimate knowledge of the relation of the diseases of animals to those of man.

"The great progress which has been made in recent years in the study of the causation of disease by microscopic investigations and laboratory work has made practeriology and colateral studies indispensible to students of comparative medicine. Such studies require thorough grounding in what may be called the fundamental subjects, such as histology and anatomy, physiology and pathological anatomy. For the study of the three first named subjects, few students in any school enjoy equal advantages either in the proficiency of the teachers or the completeness of the laboratories. While the same cannot be said for pathology, and I would here say that no benefactor who contemplates giving a helping hand for the promotion of the University can find an object more needed or more worthy than to endow a chair of pathology in such a manner as will relieve the minds of both faculties, medicine and comparative medicine, through which students both can obtain a course of instruction in this important, ay, indispensible subject, equal to that of any University on the continent.

"Since last convocation a change has taken place in the teaching staff. Professor Stewart having resigned the chair of Materia Medica, has been replaced by Professor Blackadder, who brings to his subject all his well known enthusiasm, and intends to deal with it in a most thorough manner. Professor Mills has done excellent work in his course on "Cynology, the Dog and his Diseases," a subject hitherto only taught incidentally in any college but one capable of great development, and not only has he written a special text-book on Comparative Physiology, but has also furnished his class with an admirable text-book on the Dog, which should be found in the library of every owner or lover of this most faithful friend."

At the close of the reading of the report, the following prizes were handed to the successful students by the Chairman:

Veterinary medicine and surgery—Jacob Plaskett. Anatomy —J. D. McIntyre. Diseases of cattle—J. D. McIntyre. Cynology—D. L. Bolger. Zoology—C. French. For the best general examination on all subjects—silver medal—J. D. McIntyre. Extra prizes: For the best essay read before the Veterinary Medical Association: 1st—D. L. Bolger, $15. 2nd—G. P. Wells, $10. 3rd—J. H. Seale, $5. For ths best essay read before the society for the study of comparative psychology—book—G. P. Wells. Scholarship, $50. For the highest aggregate obtained in second year subjects—Wilfred Plaskett, Scholarship, $50. For the highest aggregate obtained in first year subjects—C. French.

The degree of D. V. S. was conferred on the following students by Principal Sir Wm. Dawson, assisted by the Dean, Dr. D. McEachran :

J. D. McIntyre, R. A. Ramsay, J. Plaskett, D. L. Bolger, George Lee, G. P. Wells, J. Moffatt, A. T. Robertson. D. McNaughton, G. Gangloff, R. E. Dyer, E. Robb, J. H. Seale, S. J. Moffatt, T. B. Pote, O. C. Lofgien.

The valedictory address on behalf of the graduates was then delivered by Dr. R. A. Ramsay, of Eden Mills, Ont., who recalled many of the vissicitudes of the student life, and paid graceful tribute to the members of the faculty.

Dr. C. McEachran, D. V. S., delivered an address, reviewing the history of the twenty-six years of the college, which commenced with a course of lectures on Veterinary science, for the first time delivered during the winter of 1886 in a lecture-room on Cote Street; in 1875 the growth and importance of the school demanded a special establishment, and the present buildings were erected in that year.

He said: " The work of the present session has been eminently successful. Our students have been faithful in the performance of their various duties, and have punctually observed the strict regulations as to attendance at lectures, and corectness of conduct. No student has been suspended from his classes for absence unaccounted for, inattention or disorder: and we accordingly congratulate the class on their excellent discipline and conscientious labors. Those outside of the profession who will consult in our calendar the "Order of Lectures" will see at a glance that the time of our students is almost fully occupied, and that the degree of " Doctor of Veterinary Science" cannot possibly be gained without hard and and continuous work on the part of candidates for the honor. With the use of the Faculty's Museum, inclucing the Gadsden collection, and also that of the Medical Faculty of McGill University: enjoying too, all the privliges of the same Faculty's Physiological and Pathological Labaratories, our pupils have good opportunities for acquainting themselves thoroughly with the functions of all the different parts or organs of animals, and of studying minutely the nature of diseases, their causes and their symptoms.

In the last report of the corporation of McGill University, "The attention of the corporation has been called to the very imperfect accommodation at present possessed by the Faculty of comparative medicine and veterinary science. This professional school is of so great importance to the material interests of the Dominion that the provision of improved lecture-rooms, museum, library, etc., should commend itself as a most worthy object to those interested in our live stock and domestic animals."

Sir Wm. Dawson followed with a few words of congratulation and encouragement. He hoped that ere long fresh quarters would be assigned to this most important faculty, and expressed his good wishes for the success of the class of '92. This concluded the proceedings of the convocation.

A special session of the students was held in the library at 2.30 P. M. for the reading of the minutes, which was largely attended.

